# Outbreak of NDM-1– and OXA-181–Producing *Klebsiella pneumoniae* Bloodstream Infections in a Neonatal Unit, South Africa

**DOI:** 10.3201/eid2908.230484

**Published:** 2023-08

**Authors:** Rindidzani E. Magobo, Husna Ismail, Michelle Lowe, Wilhelmina Strasheim, Ruth Mogokotleng, Olga Perovic, Stanford Kwenda, Arshad Ismail, Manala Makua, Abram Bore, Rose Phayane, Harishia Naidoo, Tanya Dennis, Makhosazane Ngobese, Wim Wijnant, Nelesh P. Govender, for Baby GERMS-SA

**Affiliations:** National Institute for Communicable Diseases, a Division of the National Health Laboratory Service, Johannesburg, South Africa (R.E. Magobo, H. Ismail, M. Lowe, W. Strasheim, R. Mogokotleng, O. Perovic, S. Kwenda, A. Ismail, N.P. Govender);; University of the Witwatersrand, Johannesburg (O. Perovic, N.P. Govender);; National Department of Health, Pretoria (M. Makua);; Gauteng Provincial Department of Health, Johannesburg (A. Bore);; Tembisa Provincial Hospital, Johannesburg (R. Phayane, H. Naidoo, T. Dennis, M. Ngobese, W. Wijnant);; University of Cape Town, Cape Town, South Africa (N.P. Govender);; University of Exeter, Exeter, UK (N.P. Govender).

**Keywords:** healthcare-associated outbreaks, neonates, *Klebsiella pneumoniae*, carbapenem-resistance, *bla*NDM, *bla*OXA-181, whole-genome sequencing, bacteria, South Africa

## Abstract

After an increase in carbapenem-resistant *Klebsiella pneumoniae* (CRKP) bloodstream infections and associated deaths in the neonatal unit of a South Africa hospital, we conducted an outbreak investigation during October 2019–February 2020 and cross-sectional follow-up during March 2020–May 2021. We used genomic and epidemiologic data to reconstruct transmission networks of outbreak-related clones. We documented 31 cases of culture-confirmed CRKP infection and 14 deaths. Two outbreak-related clones (*bla*_NDM-1_ sequence type [ST] 152 [n = 16] and *bla*_OXA-181_ ST307 [n = 6]) cocirculated. The major clone *bla*_NDM-1_ ST152 accounted for 9/14 (64%) deaths. Transmission network analysis identified possible index cases of *bla*_OXA-181_ ST307 in October 2019 and *bla*_NDM-1_ ST152 in November 2019. During the follow-up period, 11 new cases of CRKP infection were diagnosed; we did not perform genomic analysis. Sustained infection prevention and control measures, adequate staffing, adhering to bed occupancy limits, and antimicrobial stewardship are key interventions to control such outbreaks.

Carbapenem-resistant Enterobacterales (CRE) are classified as critical priority bacterial pathogens by the World Health Organization (*1*). *Klebsiella pneumoniae* is a major cause of neonatal infections in low- and middle-income countries (*2*,*3*). In a national population-level analysis, *K. pneumoniae* accounted for 26% of invasive neonatal infections in South Africa during 2014–2019 (*4*). Resistance mechanisms to carbapenems include enzymatic inactivation, changes to outer-membrane permeability, and efflux pump upregulation (*5*). Several outbreaks of *K. pneumoniae* infection in neonatal units have been investigated in South Africa since 1992 (*6*–*11*). Some of those studies used molecular typing methods, such as multilocus sequence typing and pulsed-field gel electrophoresis (PFGE), that lack sufficient resolution to distinguish between clonal strains (*8*–*12*). Whole-genome sequencing (WGS) is a powerful tool to investigate healthcare-associated pathogens such as *K. pneumoniae* and has been widely used in combination with epidemiologic information to track outbreaks and transmission routes of pathogens (*13*–*17*).

In December 2019, the National Institute for Communicable Diseases (NICD) in South Africa was notified of 8 cases of culture-confirmed carbapenem-resistant *K. pneumoniae* (CRKP) bloodstream infections in a neonatal unit at a provincial tertiary hospital. The outbreak began with 4 cases reported in October 2019. The objectives of this investigation were to verify the existence of an outbreak; describe the antimicrobial susceptibility profiles, resistance mechanisms, and transmission dynamics of CRKP clones in circulation; and monitor the incidence of CRKP infections within 1 year of instituting outbreak interventions.

## Materials and Methods

### Hospital Setting and Surveillance

Tembisa Provincial Tertiary Hospital is located in the Tembisa township in Gauteng Province, South Africa; the township has a population size of 2.5 million. The hospital also serves the communities of Midrand and Diepsloot and as a referral site for 20 clinics in the surrounding area, spanning 3 municipalities (the cities of Johannesburg, Ekurhuleni, and Tshwane). Annually, the hospital sees >280,000 patients. The hospital facility has 840 beds in total, 704 for adults, 71 for newborn babies, and 64 for pediatric patients; the average daily admission is 150 patients. The hospital’s neonatal unit consists of wards A and B. Neonates are assigned to a section within a ward depending on age and the acuity of their condition at admission. Ward A has high-care (A1), low-care (A2), and isolation rooms; the approved bed capacity is 40. Ward B consists of a kangaroo mother care section (designed to increase skin-to-skin contact between mother and infant for preterm or low birthweight infants), a neonatal intensive care unit, a low-care unit, and a pediatric intensive care unit that is separated from the other rooms by a door. Ward B has a total of 31 beds. The intended medical staff complement during a day shift in ward A was 8 doctors, with an intended bed-to-doctor ratio of 5:1 and an intended bed-to-nurse ratio of 3:1. One medical officer was usually on night call for all the neonatal wards. Ward B had 3 doctors and 4 nurses, with an overall bed-to-staff ratio of 4.4:1.

Coincidentally, enhanced surveillance for neonatal infections as part of the Baby GERMS-SA study was conducted at this hospital during October 1, 2019–September 30, 2020 (*18*). In brief, neonates with culture-confirmed bloodstream infections or meningitis were enrolled into the surveillance program if they met the case definition (i.e., a neonate <28 days of age from whom a pathogen was isolated from blood or cerebrospinal fluid). The diagnostic laboratory submitted the corresponding bacterial and fungal isolates to the NICD for further characterization. Demographic, clinical, and outcome data were retrospectively abstracted from their imaged medical records. In addition, admissions and patient bed-days were recorded by month for the neonatal unit.

### Outbreak Investigation

A multidisciplinary investigation team consisting of members of the NICD, Infection Control Service Laboratory (National Health Laboratory Service), the Gauteng Provincial Department of Health, and the National Department of Health was assembled in January 2020. To estimate baseline rates of bloodstream infections, we obtained a line list from the NICD surveillance data warehouse for infants <6 months of age admitted to Tembisa Provincial Tertiary Hospital with blood cultures positive for any bacterial or fungal organism during January 2017–September 2019. We excluded organisms considered commensals by the US Centers for Disease Control and Prevention National Healthcare Safety Network (*19*). We regarded blood cultures with the same organism isolated within 21 days of the first positive culture as duplicates and excluded those. We defined an outbreak as an increase of >100% in the number of observed cases of CRKP infection in a month above the expected (average) number in the preceding 2 months. On March 11, 2020, before the initial investigation closeout, the World Health Organization declared the global COVID-19 pandemic (*20*). We calculated monthly bloodstream infection rates during the follow-up period, March 2020–May 2021, using the same methods used in the initial outbreak investigation.

### External Infection Prevention and Control Audit Process

During the initial investigation, we conducted an external infection prevention and control (IPC) audit in January 2020 using the National Department of Health standardized Infection Control Assessment Tool (*21*). We compiled recommendations for immediate, mid-term, and long-term interventions. A similar internal audit by the hospital response team was conducted in August 2020 to monitor improvements in adherence to IPC measures. Specific IPC measures had been implemented early after the observed increase in the number of cases. For instance, all colonized and infected babies were cohorted or isolated in separate sections of the ward. Additional cleaning of the unit was conducted several times beginning in November 2019. Hand hygiene was monitored for both staff and parents entering the unit. When the neonatal unit reached 100% bed capacity, maternity cases were rerouted to other facilities, although this intervention could not be sustained during the COVID-19 pandemic.

### Cases and Isolates

We defined a case as culture-confirmed bloodstream infection caused by *K. pneumoniae* resistant to any carbapenem (i.e., meropenem, imipenem, doripenem, or ertapenem) in an infant <6 months of age. The case definition was not restricted to neonates <28 days of age because babies who stayed in hospital beyond the neonatal period remained at risk for infection. We analyzed all isolates submitted to NICD from this hospital during October 1, 2019–February 29, 2020, including those submitted for Baby GERMS-SA surveillance. The hospital provided monthly infection reports to NICD, listing cases of CRKP infection with dates of birth, birthweights, dates of specimen collection, and outcome information. We conducted the follow-up analysis in May 2021, 18 months after the outbreak started in October 2019.

### Microbiological Analysis

We confirmed species-level identification using matrix-assisted laser desorption/ionization time-of-flight mass spectrometry (Bruker Daltonics, https://www.bruker.com) and performed antimicrobial susceptibility testing using the MicroScan WalkAway 96-plus system with the NM44 card (Siemens Healthineers, https://www.siemens-healthineers.com). We interpreted MICs for the tested antimicrobial agents according to Clinical and Laboratory Standards Institute guidelines and defined multidrug-resistant isolates as those with nonsusceptibility to >1 agent in >3 antimicrobial classes (*22*). We screened for carbapenemase genes and strain relatedness using real-time multiplex PCR and PFGE and performed WGS to determine sequence types, identify acquired antimicrobial resistance genes, and confirm presence of plasmid replicons, O antigen locus types, and K locus types. We compared core genome single-nucleotide polymorphism (SNP) distances and epidemiologic information to investigate the transmission events of CRKP in the neonatal unit (Appendix).

## Results

### Baseline, Outbreak, and Follow-Up Periods

**Figure 1:**
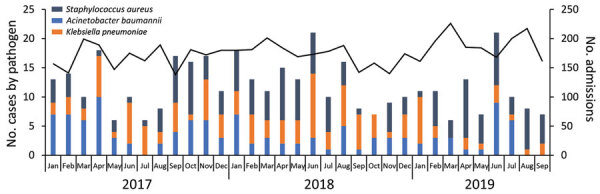
Number of cases of bloodstream infection for 3 common bacterial pathogens (n = 428) and number of admissions (n = 5,796) in the neonatal unit during baseline period, South Africa, January 2017–September 2019. Black data line indicates number of admissions. Scales for the y-axes differ substantially to underscore patterns but do not permit direct comparisons.

During January 2017–September 2019, a total of 1,771 positive blood cultures were reported from infants <6 months of age at the hospital. Of those, 864 (49%) blood cultures yielded probable pathogenic organisms; 724 were in neonatal wards A and B. We excluded 144 duplicates from the analysis. Of the remaining 580 isolates, 428 were from patients with single-isolate bloodstream infection episodes and were included in the analysis. *K. pneumoniae* accounted for 29% (122/428) of cases, *Staphylococcus aureus* 36% (153/428), and *Acinetobacter baumannii* 27% (116/428). Of the 122 cases of *K. pneumoniae* isolated during the January 2017–September 2019 baseline period, 8% (10/122) were CRKP; 5 of those cases were diagnosed in January 2019 (Figure 1).

**Figure 2:**
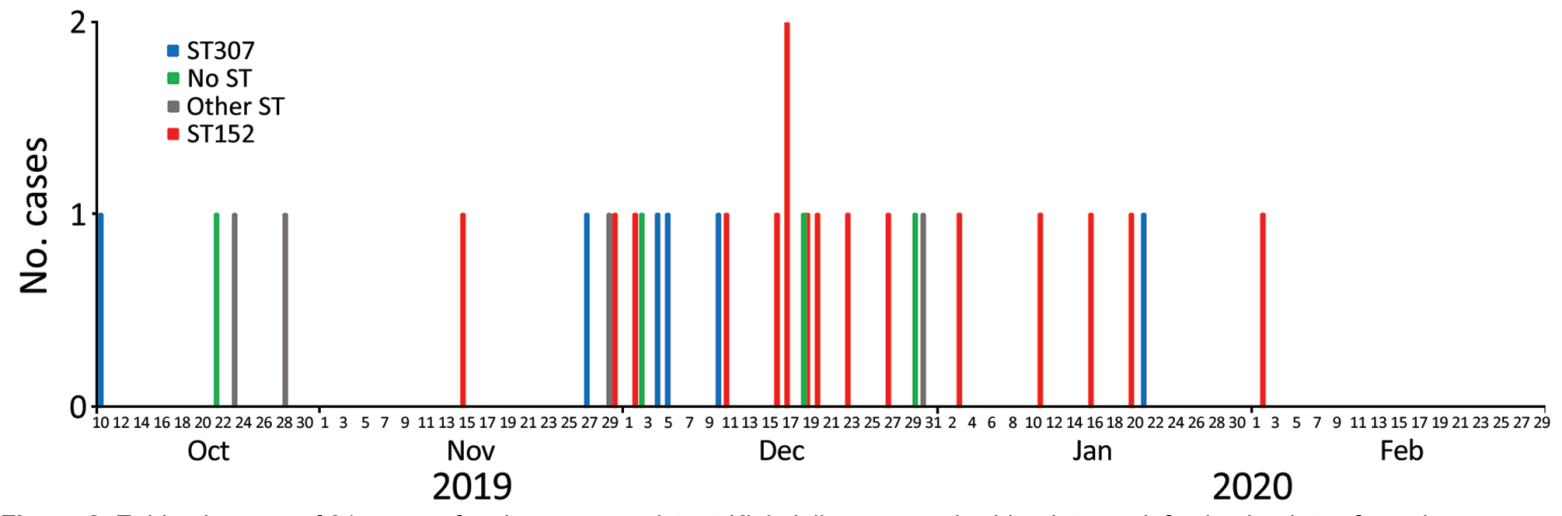
Epidemic curve of 31 cases of carbapenem-resistant *Klebsiella pneumoniae* bloodstream infection by date of specimen collection and ST of isolate during outbreak period, South Africa, October 2019–February 2020. ST, sequence type.

The number of CRKP bloodstream infection cases increased during October 1, 2019–February 29, 2020; a total of 31 cases and 15 deaths were reported. Most cases (n = 14) were reported in December 2019; in the preceding 2 months only 3 non-CRKP cases were diagnosed (average 1.5 cases/month) (Figure 2).

During the follow-up period (March 2020–May 2021), 299 positive blood cultures with probable pathogenic organisms were obtained from infants admitted to the neonatal unit (wards A and B). *K. pneumoniae* accounted for 38 of those episodes (13%); of those, 29% (n = 11) isolates were carbapenem-resistant (Appendix).

### External IPC Audit Findings and Internal Follow-Up Audit

The initial external IPC auditors found that neonatal unit ward A exceeded the approved bed capacity by 60% (64 admissions in a ward with 40 approved beds); ward A had 13 nurses per shift and a bed-to-nurse ratio of 5:1. Occupancy increased to 180% above the approved capacity during the internal audit in August 2020. Attrition occurred at a rate of 16 nurses and 13 clinical staff over a period of 7 months. Adherence to hand hygiene was 95% during the external audit and 100% during the follow-up internal audit. Hand hygiene adherence remained at >96% a year later in May 2021, maintained through a peer-monitoring system. Liquid hand soap with an antimicrobial agent, alcohol-based antiseptic, and hand lotions (aqueous cream) were initially not available for staff, but availability improved on follow-up. Alcohol-based antiseptic dispensers were available at each bed during the follow-up audit. Handwashing supplies were ordered, and stock levels were monitored. Elbow-operated taps were installed to improve quality of hand hygiene practices. Unannounced IPC audits were performed, but no direct observations were reported. The overall score for sterilizing and disinfecting instruments improved from 27% to 47% in August 2020. Policies or standard operating procedures (SOPs) were subsequently developed, and staff members signed to demonstrate their understanding of SOPs. The unit had no designated area for mixing standard intravenous fluids because of infrastructural challenges, single-dose vials were not used, and no SOP for multidose vials was in place in the ward (Appendix Table). Most external audit recommendations were implemented. For example, an integrated IPC and occupational health and safety team was established to maximize human resource capacity and budget allocations and to strengthen infection surveillance. However, some crucial initial IPC audit recommendations could not be sustained because of COVID-19 demands (e.g., diverting patients to neighboring hospitals when ward capacity was reached and converting the milk room into a kangaroo mother care unit). Although an antimicrobial stewardship committee was established, implementing its recommendations was delayed because attention and resources were diverted to the COVID-19 response.

### Isolates and Cases

**Figure 3:**
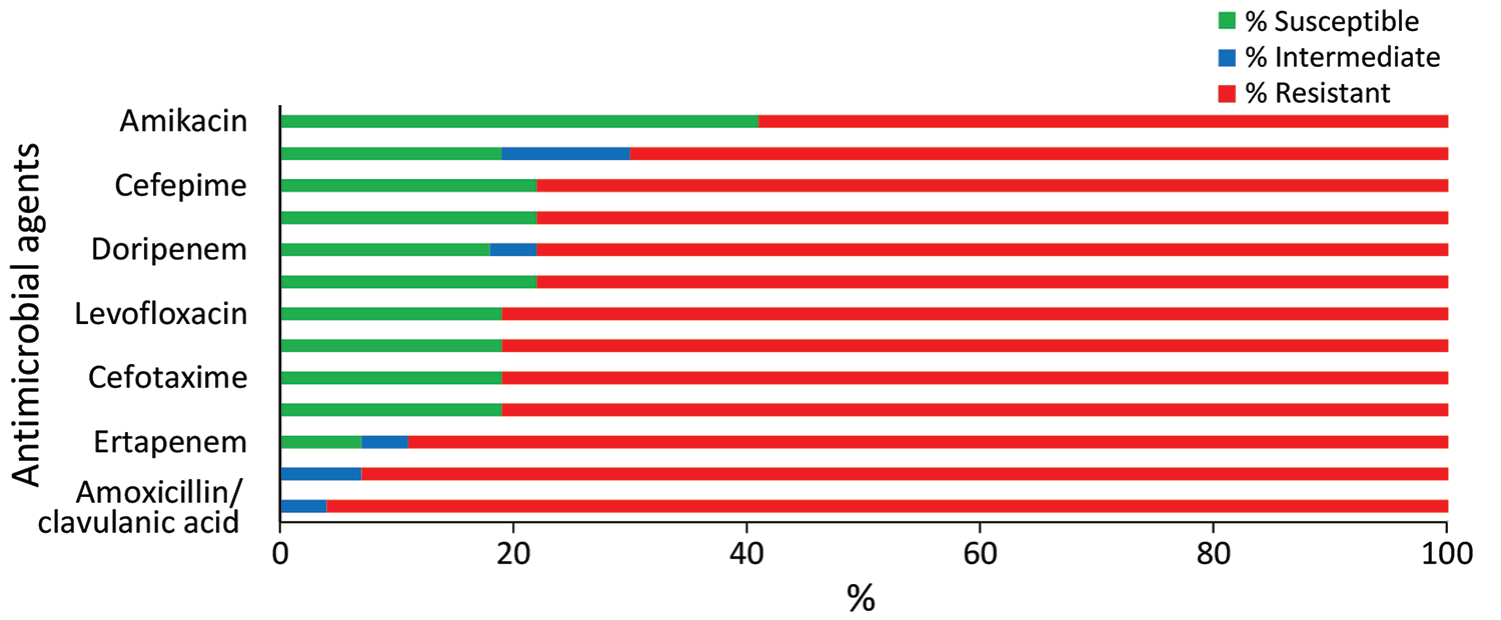
Reference laboratory antimicrobial susceptibility profiles of 27 viable *Klebsiella pneumoniae* isolates from cases of bloodstream infection during a neonatal unit outbreak, South Africa, October 2019–February 2020.

During October 2019–February 2020, a total of 31 laboratory-confirmed cases of CRKP bloodstream infections were reported in the neonatal unit. Of those, we did not have isolates for 2 cases (Figure 3), but laboratory reports indicated that these isolates were resistant to ertapenem, imipenem, and meropenem. The NICD reference laboratory received 34 isolates for the remaining 29 cases. Of those, 2 cases had 3 isolates each and 1 case had 2 isolates. The first isolate (i.e., the isolate with earliest specimen collection date) per case from 29 cases was selected for molecular characterization. Twenty-seven isolates were confirmed as *K. pneumoniae* subspecies *pneumoniae*; antimicrobial susceptibility profiling and genomic characterization was performed. We excluded 2 isolates identified as *Pseudomonas aeruginosa* that were likely contaminated during shipping.

### Antimicrobial Susceptibility

All isolates were multidrug-resistant. In total, 89% (24/27) of the isolates were resistant to ertapenem and 81% (22/27) were resistant to meropenem (Figure 3).

### Genomic Characterization

We identified 3 clonal clusters consisting of 6 sequence types from 27 isolates by using core genome maximum-likelihood phylogeny (Appendix Figure 1). Sequence type (ST) 152 accounted for 59% (16/27) of the cases, followed by ST307 (6/27; 22%) and ST17 (2/27; 7%); ST25, ST45, and ST297 each accounted for a single case (1/27; 4%). We found that 94% (26/27) of the isolates carried carbapenemase genes. All isolates within ST152 clone harbored the *bla*_NDM-1_ gene and contained ColRNAI, *Inc*FIB(K), *Inc*FIB(pB171), and *Inc*FII(Yp) plasmid incompatibility groups. Further analysis revealed that the *bla*_NDM-1_ gene was carried on the *Escherichia coli* B171 plasmid pB171 (accession no. AB024946). Phenotypic resistance to third and fourth generation cephalosporins was confirmed by the presence of extended-spectrum β-lactamases, such as *bla*_CTX-M-15_ and other β-lactamase genes (*bla*_TEM-1_ and *bla*_SHV-1_) (Appendix Figure 1).

The gene *bla*_OXA-181_ was present in all isolates belonging to ST307. Three isolates carrying the *bla*_OXA-181_ gene coharbored the *bla*_OXA-48_ gene. All isolates within *bla*_OXA-181_ ST307 clone shared ColKP3, IncFIB(Mar), and IncX3 plasmid incompatibility groups. Plasmid analysis showed that *bla*_OXA-181_ gene in ST307 clone isolates was carried on *K. pneumoniae* KP3 plasmid KP3-A (accession no. JN205800) (Appendix Figure 1).

### Clinical Characteristics of 26 WGS-Confirmed Cases of CRKP

Of the 26 WGS-confirmed cases of CRKP, 67% (18/26) of the infants were boys; median age was 7 days (interquartile range 3–17 days). Birthweights ranged from 760 to 3,200 g; the median weight was 1,100 g (interquartile range 970–1595 g). Four of the 26 infants required resuscitation at birth. Invasive devices were inserted in all infants. Two infants had a record of underlying abnormalities (congenital anemia and neonatal seizures). Just over half (54% [14/26]) of infants died in hospital. Of these, 64% (9/14) had cultured isolates belonging to the ST152 clone (Table).

**Table:** Clinical characteristics of WGS-confirmed cases of carbapenem-resistant *Klebsiella pneumoniae* bloodstream infection in newborns admitted to the neonatal unit of a tertiary care hospital, South Africa, October 2019–February 2020*

Pt no.	Age, d/sex	Weight at birth, g	Gestational age, wks	Ward†	Other pathogens	Outcome	Isolate	Collection date	ST	K type	O type
Pt 1	13/F	1,300	32	A		Transferred	BG32	2019 Oct 10	307	KL102	O2afg
Pt 2	31/M	1,000	28	A		Discharged	BG113	2019 Oct 23	17	KL25	O5
Pt 3	0/M	3,200	42	A		Discharged	BG265	2019 Oct 28	17	KL25	O5
Pt 4	7/M	1,000	28	A1, B	*Acinetobacter baumannii*	Died	BG314	2019 Nov 15	152	KL149	O4
Pt 5	20/F	890	28	A		Discharged	BG315	2019 Nov 27	307	KL102	O2afg
Pt 6	10/F	1,360	27	A1, A2, B	*Enterobacter cloacae*	Died	BG313	2019 Nov 29	297	KL158	O1
Pt 7	2/M	1,000	NR	A	*Acinetobacter baumannii*	Died	BG263	2019 Nov 30	152	KL149	O4
Pt 8	5/F	1,700	34	A		Died	BG264	2019 Dec 2	152	KL149	O4
Pt 9	17/M	1,100	NR	B		Died	BG259	2019 Dec 4	307	KL102	O2afg
Pt 10	9/M	2,690	38	A		Died	BG258	2019 Dec 5	307	KL102	O2afg
Pt 11	23/M	1,100	28	B		Died	BG254	2019 Dec 10	307	KL102	O2afg
Pt 12	7/M	840	26	A1, isolation		Discharged	BG255	2019 Dec 11	152	KL149	O4
Pt 13	1/M	NR	NR	A		Discharged	BG222	2019 Dec 16	152	KL149	O4
Pt 14	21/M	1,570	34	A		Discharged	BG218	2019 Dec 17	152	KL149	O4
Pt 15	4/F	3,050	39	A		Died	BG213	2019 Dec 17	152	KL149	O4
Pt 16	19/F	970	NR	A	*Enterobacter cloacae*	Died	BG223	2019 Dec 19	152	KL149	O4
Pt 17	7/F	800	29	A		Died	BG219	2019 Dec 20	152	KL149	O4
Pt 18	3/M	1,300	29	A1		Died	BG215	2019 Dec 23	152	KL149	O4
Pt 19	3/F	1,700	34	A		Discharged	BG214	2019 Dec 27	152	KL149	O4
Pt 20	6/M	810	29	A2		Died	BG442	2019 Dec 30	25	KL2	O1
Pt 21	0/F	1,670	33	A		Discharged	BG272	2020 Jan 3	152	KL149	O4
Pt 22	0/M	1,100	30	A1, isolation		Discharged	BG271	2020 Jan 11	152	KL149	O4
Pt 23	8/M	970	30	A1, isolation, B		Discharged	BG460	2020 Jan 16	152	KL149	O4
Pt 24	12/M	1,240	28	A1, A2, B		Died	BG316	2020 Jan 20	152	KL149	O4
Pt 25	13/M	1,010	27	A1, isolation, B		Discharged	BG317	2020 Jan 21	307	KL102	O2afg
Pt 26	6/M	925	29	A1, B		Died	BG449	2020 Feb 2	152	KL149	O4

*The specimen type used for all patients was blood culture. NR, not recorded; Pt, patient; ST, sequence type; WGS, whole-genome sequencing. †Wards A1, A2, and Isolation are sections (cubicles) within ward A with a total approved bed capacity of 40.

### Genomic and Epidemiologic Links among Outbreak-Associated Isolates

**Figure 4:**
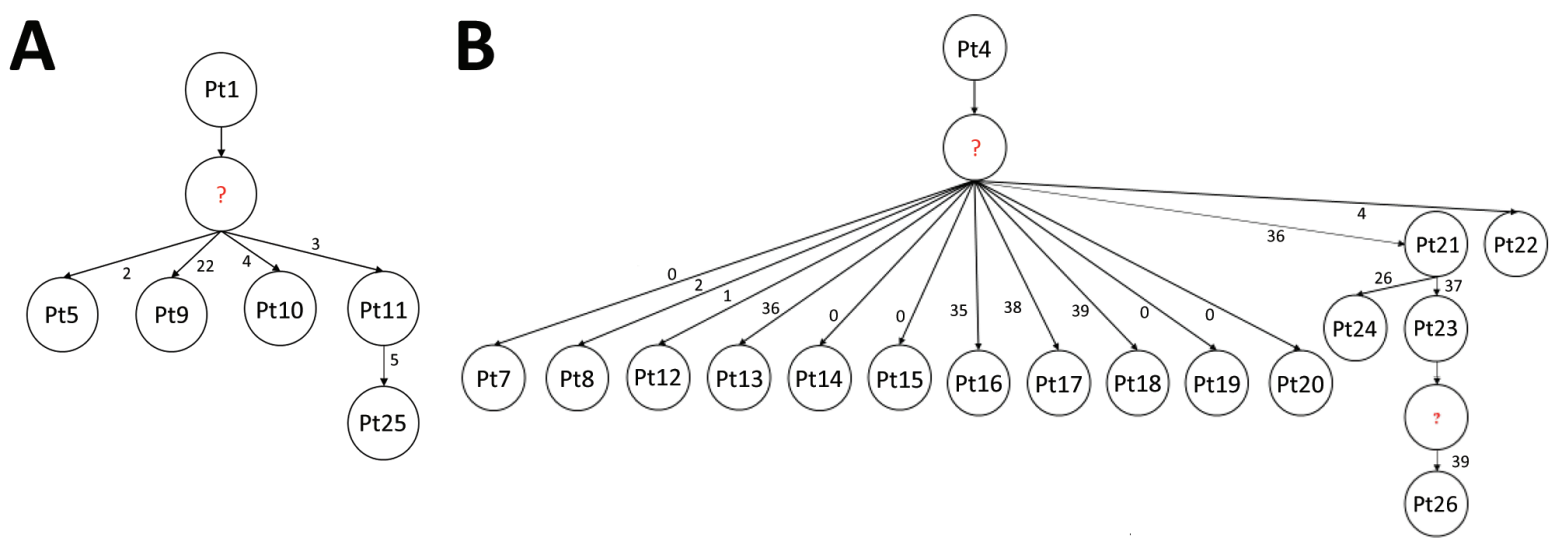
Transmission networks among 22 patients with outbreak-associated *bla*_OXA-181_ sequence type 307 (A) and *bla*_NDM-1_ sequence type 152 (B) clones of *Klebsiella pneumoniae* isolated from a neonatal unit during outbreak, South Africa, October 2019–February 2020. Question marks denote missing isolates; numbers along branches indicate number of single-nucleotide polymorphisms between index isolate and other isolates.

The first case (Pt1) of CRKP BSI *bla*_OXA-181_ ST307 clone was confirmed on October 10, 2019 in ward A. The *bla*_OXA-181_ ST307 was responsible for 4 more cases (2 each in wards A and B) in late November and during the first week of December 2019. The last case belonging to *bla*_OXA-181_ ST307 clone was identified in January 2020. The transmission network revealed that Pt1 was a possible index case. Although this patient was later transferred to an academic hospital for further treatment, this clone continued to disseminate within the neonatal unit. Five more cases were detected with the transmission stemming from the intermediate host with SNP differences of <10 among the clonal isolates (Figure 4, panel A).

The first case-patient (Pt4) of the major outbreak-related *bla*_NDM-1_ ST152 clone was diagnosed on November 15, 2019, in ward A of the neonatal unit, followed by another case (Pt7) 15 days later in ward A. During December 11, 2019–February 2, 2020, a total of 14 more cases were isolated in the neonatal unit. Isolates from Pt14 and Pt17 were not considered part of the outbreak because they had >30 SNPs compared to other isolates. No further cases caused by the *bla*_NDM-1_ ST152 clone were diagnosed after February 2020. Transmission network analysis identified Pt4 as the index case for the ST152 clone outbreak. However, Pt4 died on November 16, 2019, ten days before Pt7 was admitted, suggesting the role of an intermediate host or environmental source in the dissemination of *bla*_NDM-1_ ST152 clone in the neonatal unit. Pt8’s infection was diagnosed 2 days after the diagnosis of Pt7. Pt8 died a day after diagnosis. No further transmission from Pt7 or Pt8 to other patients could be established. Nine patients (Pt12–Pt19 and Pt22) in whom transmission stemmed directly from Pt4 were potentially infected through an intermediate source. Further dissemination of the ST152 clone was observed between Pt21, Pt23, and Pt26 in January 2020. These patients were admitted to ward A but moved between ward A and ward B. The transmission network revealed 2 possible events: first, from Pt21 to Pt24, who died a day after diagnosis; and second, that transmission occurred from Pt21 to Pt23, then from Pt23 to Pt26. However, Pt26 was admitted to ward A, 15 days after Pt23 was discharged from the hospital, suggesting the role of an intermediate in the spread of *bla*_NDM-1_ ST152 clone (Figure 4, panel B).

## Discussion

We describe the successful integration of genomic and epidemiologic data in tracking an outbreak of CRKP infections in the neonatal unit of a South Africa hospital. The number of CRKP bloodstream infections increased substantially in the neonatal unit beginning in October 2019. Two outbreak-related clones (*bla*_NDM-1_ ST152 and *bla*_OXA-181_ ST307) cocirculated. The major outbreak clone *bla*_NDM-1_ ST152 accounted for >60% of deaths. Fine transmission networks identified possible index cases of *bla*_OXA-181_ ST307 and *bla*_NDM-1_ ST152 clones. The spread and dissemination of these clones from patient to patient might have been enabled by healthcare workers or environmental or fomite contamination, resulting from breaches in IPC measures.

Reports of CRE infections in Africa have increased markedly; the most commonly reported carbapenemases are *bla*_NDM-1_ and *bla*_OXA-48_ and variants (*23*–*27*). In this study, *K. pneumoniae* carrying *bla*_NDM-1_, *bla*_OXA-48_, and *bla*_OXA-181_ were dominant in the neonatal unit, which was consistent with previous reports (*23*–*27*). The *bla*_NDM_ gene was first described in South Africa in an *Enterobacter cloacae* strain in 2011 (*28*). Recent data from South Africa sentinel surveillance of CRE bloodstream infections have shown a change in the distribution of carbapenemase genes (*29*). Previously, *bla*_NDM_ was the most commonly detected gene in Enterobacterales, followed by *bla*_OXA-48_ (*25*). However, beginning in 2016, *bla*_OXA-48_ and variants began to dominate, followed by *bla*_NDM_ (*29*). This change is worrisome because *bla*_OXA-48_ and variants are more difficult to identify using phenotypic laboratory methods. Most outbreaks reported in South Africa are also associated with *bla*_OXA-48_ and variants (*30*–*32*). The emergence of *bla*_NDM-1_ *K. pneumoniae* as a cause of outbreaks is concerning because once inserted into drug-resistant plasmids, virulence determinants render these strains highly resistant, virulent, and easily transmissible regardless of the clone (*33*).

The first laboratory-confirmed outbreak of *bla*_OXA-181_ *K. pneumoniae* in South Africa was described in 2015 from a hematology unit in Cape Town (*30*). After that, Strydom et al. (*31*) identified *K. pneumoniae* superclone ST307 carrying *bla*_OXA-181_ on a self-transmissible plasmid IncX3 (p72_X3_OXA181) as the source of a CRKP outbreak that spread across multiple wards in a Pretoria hospital during September 2015–December 2016. Furthermore, *bla*_OXA-181_ was endemic among patients colonized with *K. pneumoniae* in a KwaZulu-Natal hospital intensive care unit, and the spread was enabled by plasmid replicon *E. coli* p010-B-OXA181 (*32*). A combination of genomic and epidemiologic data was used either to track outbreaks or reconstruct transmission events that occurred during an outbreak (*14*,*16*,*34**–**36*). In our study, *bla*_NDM-1_ and *bla*_OXA-181_ *K. pneumoniae* strains were responsible for outbreaks that occurred simultaneously in the neonatal unit. The resistant genes were spread by transmissible plasmids *E. coli* B171 plasmid pB171 (*bla*_NDM-1_) and *K. pneumoniae* KP3 plasmid KP3-A (*bla*_OXA-181_). A recent report describing an outbreak of CRKP bloodstream infections in a neonatal unit in another South Africa hospital showed that *bla*_OXA-48_ and variants *K. pneumoniae* were responsible for the outbreak (*11*). Although the main objectives of the outbreak investigation by Essel et al. (*11*) were to confirm the outbreak and assess the IPC program, the molecular typing technique used has low discriminatory power to distinguish genetically related isolates. Lowe et al. (*37*) documented the rapid spread of ST307 clone carrying *bla*_OXA-181_ in >40 hospitals across 3 provinces in South Africa, highlighting the critical need for more enhanced genomic surveillance of *K. pneumoniae* ST307 super clone in healthcare settings.

Given the rapid transfer and acquisition of endemic carbapenemase genes, IPC measures are critical to preventing and managing outbreaks. Both IPC audits flagged <50% adherence to instrument sterilization and disinfection procedures. Therefore, breaches in aseptic techniques during invasive medical device insertion or maintenance practices might have been involved in the causal pathway to neonatal bacteremia. Inadequate staffing, exceeded bed capacity, aging and undermaintained hospital infrastructure, and general lack of institutional support for IPC and antimicrobial stewardship initiatives also contribute to the spread of healthcare-associated infections (*38*). Dramowski et al. presented a framework for prevention of healthcare-associated infections in neonates and children, which highlighted the need for a nationally endorsed prevention strategy to ensure that children in South Africa receive safe and high-quality care (*39*).

The strength of this study was involvement of multiple stakeholders who enabled different segments of the outbreak investigation. Genomic and epidemiologic data were used to confirm the existence of the outbreak, identify the sources of the outbreak, and reconstruct probable transmission events. The first limitation of our study is that we conducted a search for cases of bloodstream infection among infants <6 months of age through a laboratory audit, but we did not extend this search to the rest of the hospital. Second, not all isolates from laboratory-confirmed CRKP cases were available for molecular characterization. Finally, genetic links among isolates from infected neonates and contaminated environment, fomites, or colonized healthcare workers could not be established because we did not perform contemporary environmental sampling or a colonization survey.

In conclusion, a combination of high-resolution WGS and epidemiologic data enabled a detailed description of this healthcare-associated infection outbreak in a neonatal unit and established transmission links. Continued monitoring of pathogens carrying endemic carbapenemases is necessary to prevent further reemergence of outbreaks. IPC measures complemented with adequate staffing levels, adherence to bed occupancy limits, improved neonatal unit infrastructure, and antimicrobial stewardship are key to sustainably reducing neonatal healthcare-associated infections.

Appendix Additional information about outbreak of NDM-1– and OXA-181–producing *Klebsiella pneumoniae* bloodstream infections in a neonatal unit, South Africa.
