# Supplementary material for: Outbreak of NDM-1– and OXA-181–Producing Klebsiella pneumoniae Bloodstream Infections in a Neonatal Unit, South Africa
Source: Emerg Infect Dis. 2023 Aug;29(8):1531–9. doi: 10.3201/eid2908.230484 (PMC10370860; doi:10.3201/eid2908.230484)
Supplement: Appendix — Additional information about outbreak of NDM-1– and OXA-181–producing Klebsiella pneumoniae bloodstream infections in a neonatal unit, South Africa. [file 23-0484-Techapp-s1.pdf]

Article DOI: https://doi.org/10.3201/eid2908.230484

EID cannot ensure accessibility for supplementary materials supplied by authors. Readers who have difficulty accessing supplementary content should contact the authors for assistance.

Outbreak of NDM-1- and OXA-181-Producing *Klebsiella pneumoniae* Bloodstream Infections in a Neonatal Unit, South Africa

Appendix

Ethics and approvals

Ethics approval for NICD outbreak investigations was granted by the University of the Witwatersrand Human Research Ethics (Medical) Committee (M210752). Ethical approval and permission to conduct Baby GERMS-SA surveillance and this outbreak investigation were granted by the University of Witwatersrand Human Research ethics committee (M190320), the Gauteng Department of Health, and hospital management.

Additional Methods

Real-time polymerase chain reaction for carbapenemase genes

Genomic DNA from carbapenem-resistant *K. pneumoniae* isolates was extracted using a crude boiling method. The presence of six carbapenemase genes (KPC, OXA-48, NDM, GES, IMP and VIM) was detected using a commercial multiplex real-time polymerase chain reaction (PCR) assay (LightMix Modular Carbapenemases) on the LightCycler II 480 instrument (Roche Diagnostics, Germany).

Pulsed-field gel electrophoresis

Pulsed-field gel electrophoresis (PFGE) was performed to determine the genetic relatedness of *K. pneumoniae* isolates from 1 October 2019. *Salmonella enterica* serotype

Branderup H9812 was used as a molecular size marker and control. Agarose plugs for both *K. pneumoniae* isolates and the control strain were digested with *Xba*I (New England Biolabs, UK) at 37°C for 4 hours. The digested plugs were separated on the BioRad CHEF DR III system (Bio-rad laboratories, USA). The switch time was set at 2.2 seconds to 63.8 seconds. The gel ran at a voltage of 6V/cm for 19 h (14°C) at an angle 120°. Cluster analysis was done using BioNumerics (GelCompar II, Applied Math, UK). Isolates were considered genetically related if banding patterns showed ≥80% similarity and were assigned as pulsotypes.

PFGE banding patterns on all isolates collected during the outbreak period showed that isolates harbored the NDM gene were genetically related and clustered together into one major pulse group. Isolates that harbored the OXA-48 and variant gene were distinguishable from isolates that harbored the NDM gene and clustered into two minor pulsogroups. The additional four isolates from cases detected during the follow-up period had different PFGE banding patterns and could not be associated or linked to the two outbreak clusters.

Whole genome sequencing, genome annotation, single nucleotide polymorphisms and cluster analysis

Genomic DNA was extracted using QIAamp DNA mini kit (Qiagen, Texas, USA) following the manufacturer's instructions. The purity and concentrations were checked using the Nanodrop 2000 (ThermoFisher Scientific, Waltham, MA, USA) and Qubit 4 fluorometer (ThermoFisher Scientific) respectively. Multiplexed, paired-end libraries were prepared using the Illumina DNA Prep kit (Illumina, San Diego, California, USA). Sequencing was performed on Illumina NextSeq 550 platform (Illumina) (2x 150bp) with 100x coverage at the NICD Sequencing Core Facility. WGS-based strain typing was performed using Jekesa pipeline (v1.0; https://github.com/stanikae/jekesa). Initially, the generated sequence raw reads were filtered (Q>30 and length >50 bp) using Trim Galore (v0.6.2; https://github.com/FelixKrueger/TrimGalore) and de novo assembly and optimization of the contigs was done using SPAdes v3.13 and Shovill (v1.1; https://github.com/tseemann/shovill), respectively. Species prediction, MLST, acquired antimicrobial resistance genes, and presence of plasmid replicons, O antigen locus types, and K locus types were determined using Kleborate (https://github.com/katholt/Kleborate). Core genome alignments were generated using Snippy (v4.6; https://github.com/tseemann/snippy), with *K. pneumoniae* subspecies *pneumoniae* HS11286 (accession number: CP003200) as reference. Recombination regions were detected and

removed using Gubbins (*1*), and core single-nucleotide polymorphism (SNP) alignments were obtained using snp-sites (*2*). Maximum-likelihood phylogenetic trees were generated with IQ-TREE 2 (*3*) based on the generalized time reversible (GTR) model with ascertainment bias correction. Microreact (https://microreact.org/) was used to edit and visualize the trees.

To investigate the transmission events of CR-*K. pneumoniae* in the neonatal unit, we compared the core genome SNP distances along with epidemiologic information such as ward, date of positive blood culture, neonatal ward admission date, final outcome and outcome date. Transmission network reconstruction was performed using seqTrack function implemented in the adgenet package v2.1.3 in R (*4*). For seqTrack analysis, 11 SNPs were arbitrarily used as a cutoff to identify isolates likely to be related to the outbreak (*5*–*8*).

References

1. Croucher NJ, Page AJ, Connor TR, Delaney AJ, Keane JA, Bentley SD, et al. Rapid phylogenetic analysis of large samples of recombinant bacterial whole genome sequences using Gubbins. Nucleic Acids Res. 2015;43:e15. PubMed https://doi.org/10.1093/nar/gku1196

2. Page AJ, Taylor B, Delaney AJ, Soares J, Seemann T, Keane JA, et al. SNP-sites: rapid efficient extraction of SNPs from multi-FASTA alignments. Microb Genom. 2016;2:e000056. **PMID 28348851**

3. Minh BQ, Schmidt HA, Chernomor O, Schrempf D, Woodhams MD, von Haeseler A, et al. IQ-TREE 2: new models and efficient methods for phylogenetic inference in the genomic era. Mol Biol Evol. 2020;37:1530–4. PubMed https://doi.org/10.1093/molbev/msaa015

4. Nordmann P, Poirel L. The difficult-to-control spread of carbapenemase producers among Enterobacteriaceae worldwide. Clin Microbiol Infect. 2014;20:821–30. PubMed https://doi.org/10.1111/1469-0691.12719

5. Onori R, Gaiarsa S, Comandatore F, Pongolini S, Brisse S, Colombo A, et al. Tracking nosocomial *Klebsiella pneumoniae* infections and outbreaks by whole-genome analysis: small-scale Italian scenario within a single hospital. J Clin Microbiol. 2015;53:2861–8. PubMed https://doi.org/10.1128/JCM.00545-15

6. Spencer MD, Winglee K, Passaretti C, Earl AM, Manson AL, Mulder HP, et al. Whole genome sequencing detects inter-facility transmission of carbapenem-resistant *Klebsiella pneumoniae*. J Infect. 2019;78:187–99. PubMed https://doi.org/10.1016/j.jinf.2018.11.003

7. Stoesser N, Giess A, Batty EM, Sheppard AE, Walker AS, Wilson DJ, et al. Genome sequencing of an extended series of NDM-producing *Klebsiella pneumoniae* isolates from neonatal infections in a Nepali hospital characterizes the extent of community- versus hospital-associated transmission in an endemic setting. Antimicrob Agents Chemother. 2014;58:7347–57. PubMed https://doi.org/10.1128/AAC.03900-14

8. Haller S, Eller C, Hermes J, Kaase M, Steglich M, Radonic A, et al. What caused the outbreak of ESBL- producing Klebsiella pneumoniae in a neonatal intensive care unit, Germany 2009 to 2012? Reconstructing transmission with epidemiological analysis and whole-genome sequencing. BMJ Open. 2015;5:e007397. **PMID 25967999**

Appendix Table. Summary of the infection prevention and control audit findings

Key assessed areas by module section	External audit (January 2020 score), % (category*)	Internal audit (August 2020) score, % (category*)
Infection prevention programs	81 (A)	91 (A)
Infection control regulations and authority	84 (A)	94 (A)
Infection control committee	81 (A)	88 (A)
Key infection control personnel	60 (B)	80 (A)
Infection control education programs	100 (A)	96 (A)
Outbreak investigation and nosocomial infection surveillance	80 (A)	98 (A)
Hand hygiene practices	95 (A)	100 (A)
Hand hygiene policies	89 (A)	100 (A)
Hand hygiene practices in the clinical areas of neonatal ward	100 (A)	100 (A)
Isolation and standard precautions	86 (B)	82 (A)
Isolation policies and precautions	71 (B)	63 (B)
Supplies for isolation precautions	100 (A)	100 (A)
Intensive care unit staffing	70 (B)	65 (B)
Staffing	100 (A)	60 (A)
General practices in ICU	50 (B)	54 (A)
Mechanical ventilation	100 (A)	94 (A)
Prophylaxis and monitoring	31 (C)	51 (A)
Intravenous catheter and intravenous fluids and medication	48 (C)	71 (B)
Use of intravenous catheter	56 (B)	75 (B)
Preparation of intravenous fluids and medications	40 (C)	66 (B)
Sterilization and disinfection-equipment and IV fluids	51 (B)	58 (B)
Decontamination and cleaning of instruments and equipment	58 (B)	61 (B)
Sterilization and disinfection of instruments and equipment	27 (C)	47 (C)
Chemical sterilization	67 (B)	67 (B)
Pharmacy	63 (B)	61 (B)
Pharmacy key personnel	100 (A)	100 (A)
Pharmacy services	74 (B)	74 (B)
Antibiotic control program	53 (B)	44 (B)
Antibiotic monitoring and reporting	25 (C)	25 (B)

*Each key area was scored based on level of adherence: A (≥80%), B (50%–80%), and C (<50%).
Seven key areas of the ICAT were assessed: IPC program, hand hygiene practices, isolation and standard practices, intensive care unit staffing, intravenous catheter, and intravenous fluids and medications, sterilization and disinfection of intravenous sites, and pharmacy. Each key area was scored based on the level of adherence: A (≥80%), B (50%–80%), and C (<50%).

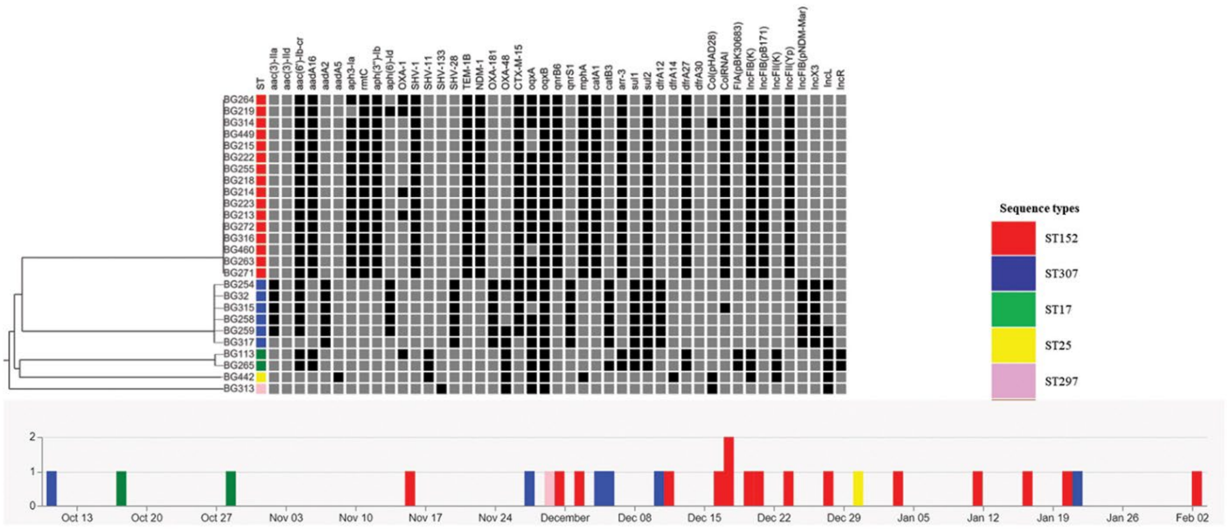

Appendix Figure 1. Maximum-likelihood phylogenetic tree based on core-genome single nucleotide polymorphisms showing clustering and antimicrobial-resistance genes of viable carbapenem-resistant *Klebsiella pneumoniae* isolates from cases of bloodstream infection admitted to neonatal unit, South Africa, October 2019–February 2020 (outbreak period), n = 26. Grey color indicates absent, black color indicates present.

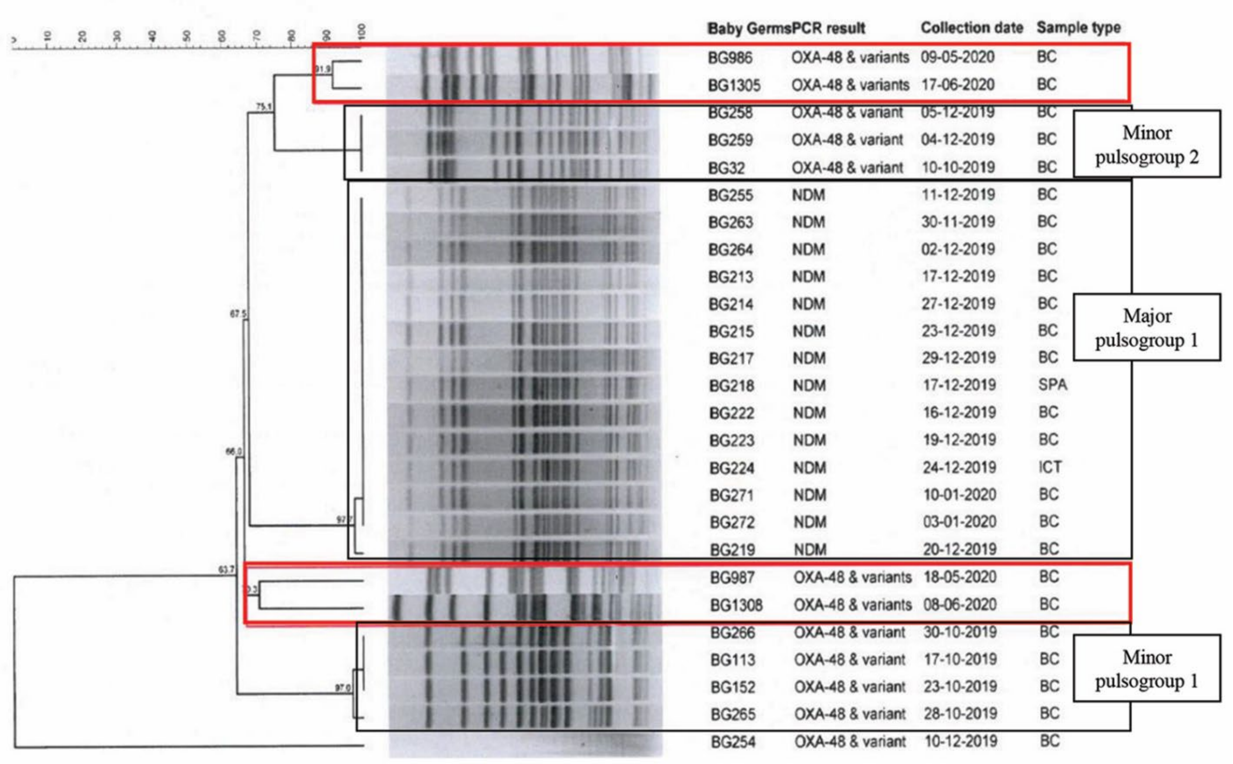

Appendix Figure 2. Pulsed-field gel electrophoresis banding patterns of clinical *K. pneumoniae* isolates collected during an outbreak and follow-up (boxed in red) in the neonatal unit
